# Reducing Side Effects of Hiding Sensitive Itemsets in Privacy Preserving Data Mining

**DOI:** 10.1155/2014/235837

**Published:** 2014-04-10

**Authors:** Chun-Wei Lin, Tzung-Pei Hong, Hung-Chuan Hsu

**Affiliations:** ^1^Innovative Information Industry Research Center (IIIRC), School of Computer Science and Technology, Harbin Institute of Technology Shenzhen Graduate School, Shenzhen 518055, China; ^2^Shenzhen Key Laboratory of Internet Information Collaboration, School of Computer Science and Technology, Harbin Institute of Technology Shenzhen Graduate School, Shenzhen 518055, China; ^3^Department of Computer Science and Information Engineering, National University of Kaohsiung, Kaohsiung 811, Taiwan; ^4^Department of Computer Science and Engineering, National Sun Yat-Sen University, Kaohsiung 804, Taiwan

## Abstract

Data mining is traditionally adopted to retrieve and analyze knowledge from large amounts of data. Private or confidential data may be sanitized or suppressed before it is shared or published in public. Privacy preserving data mining (PPDM) has thus become an important issue in recent years. The most general way of PPDM is to sanitize the database to hide the sensitive information. In this paper, a novel hiding-missing-artificial utility (HMAU) algorithm is proposed to hide sensitive itemsets through transaction deletion. The transaction with the maximal ratio of sensitive to nonsensitive one is thus selected to be entirely deleted. Three side effects of hiding failures, missing itemsets, and artificial itemsets are considered to evaluate whether the transactions are required to be deleted for hiding sensitive itemsets. Three weights are also assigned as the importance to three factors, which can be set according to the requirement of users. Experiments are then conducted to show the performance of the proposed algorithm in execution time, number of deleted transactions, and number of side effects.

## 1. Introduction

With the rapid growth of data mining technologies in recent years, useful information can be easily mined to aid mangers or decision-makers for making efficient decisions or strategies. The derived knowledge can be simply classified into association rules [1–5], sequential patterns [6–8], classification [9, 10], clustering [11, 12], and utility mining [13–16], among others. Among them, association-rule mining is the most commonly used to determine the relationships of purchased items in large datasets.

Traditional data mining techniques analyze database to find potential relations among items. Some applications require protection against the disclosure of private, confidential, or secure data. Privacy preserving data mining (PPDM) [17] was thus proposed to reduce privacy threats by hiding sensitive information while allowing required information to be mined from databases. Privacy information includes some personal or confidential information in business, such as social security numbers, home address, credit card numbers, credit ratings, purchasing behavior, and best-selling commodity. In PPDM, data sanitization is generally used to hide sensitive information with the minimal side effects for keeping the original database as authentic as possible. The intuitive way of data sanitization to hide sensitive information is directly to delete sensitive information from amounts of data. Three side effects of hiding failure, missing cost, and artificial cost are then generated in data sanitization process but most approaches are designed to partially evaluate the side effects. Infrequent itemset is, however, not considered in the evaluation process, thus raising the probability of artificial itemsets caused. Besides, the differences between the minimum support threshold and the frequencies of the itemsets to be hidden are not considered in the above approaches.

In this paper, a hiding-missing-artificial utility (HMAU) algorithm is proposed for evaluating the processed transactions to determine whether they are required to be deleted for hiding sensitive itemsets by considering three dimensions as hiding failure dimension (HFD), missing itemset dimension (MID), and artificial itemset dimension (AID). The weight of each dimension in evaluation process can be adjusted by users. Experimental results showed that the proposed HMAU algorithm has good performance in execution time and the number of deleted transactions. Besides, the proposed algorithm can thus generate minimal side effects of three factors compared to the past algorithm for transaction deletion to hide the sensitive itemsets.

This paper is organized as follows. Some related works are reviewed in Section 2, including the data mining techniques, the privacy preserving data mining, and the evaluated criteria of PPDM. The proposed HMAU algorithm to hide the sensitive itemsets for transaction deletion is stated in Section 3. An illustrated example of the proposed HMAU algorithm is given in Section 4 step by step. Experiments are conducted in Section 5. Conclusion and future works are mentioned Section 6.

## 2. Review of Related Works

In this section, privacy preserving data mining (PPDM) techniques and evaluated criteria of PPDM are respectively reviewed.

### 2.1. Privacy Preserving Data Mining Techniques

Data mining is used to extract useful rules from large amounts of data. Agrawal and Srikant proposed Apriori algorithm to mine association rules in two phases to firstly generate the frequent itemsets and secondly derive the association rules [3]. Han et al. then proposed the Frequent-Pattern-tree (FP-tree) structure for efficiently mining association rules without generation of candidate itemsets [18]. The FP-tree was used to compress a database into a tree structure which stored only large items. It was condensed and complete for finding all the frequent patterns. The construction process was executed tuple by tuple, from the first transaction to the last one. After that, a recursive mining procedure called FP-Growth was executed to derive frequent patterns from the FP-tree.

Through various data mining techniques, information can thus be efficiently discovered. The misuse of these techniques may, however, lead to privacy concerns and security problems. Privacy preserving data mining (PPDM) has thus become a critical issue for hiding private, confidential, or secure information. Most commonly, the original database is sanitized for hiding sensitive information [19–21].

In data sanitization, it is intuitive to directly delete sensitive data for hiding sensitive information. Leary found that data mining techniques can pose security and privacy threats [22]. Amiri proposed the aggregate, disaggregate, and hybrid approaches to, respectively, determine whether the transactions or the items are to be deleted for hiding sensitive information [23]. The approaches considered the ratio of sensitive itemsets to nonsensitive frequent itemsets to evaluate the side effects of hiding failures and missing itemsets. Oliveira and Zaïane designed the sliding window algorithm (SWA) [24], in which the victim item with the highest frequency in the sensitive rules related to the current sensitive transaction is selected. Victim items are removed from the sensitive transaction until the disclosure threshold equals 0. Hong et al. proposed a lattice-based algorithm to hide the sensitive information through itemset deletion by a lattice structure to speed up the sanitization process [25]. All the sensitive itemsets are firstly used to build the lattice structure. The sensitive itemsets are then gradually deleted bottom-up form the lowest levels to the highest ones until the frequencies of the sensitive itemsets are lower than the minimum support threshold. Different strategies for hiding sensitive itemsets are still designed in progress to find better results considering of side effects and the dissimilarity of database [21, 26–30].

### 2.2. Evaluation Criteria

**Figure 1:**
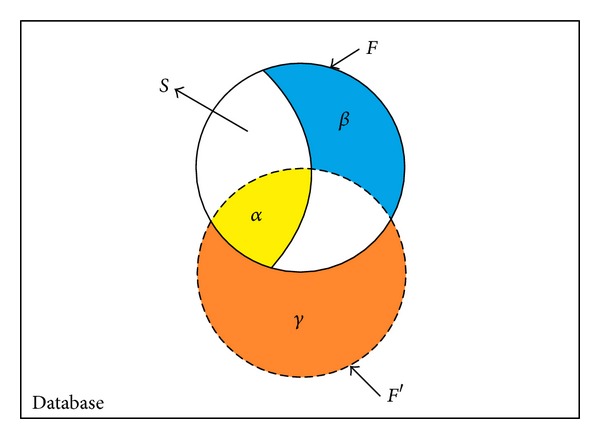
Relationship between the side effects and mined rules of the original database and sanitized one.

In data sanitization, the primary goal is to hide the sensitive information with minimal influences on databases. Three side effects of hiding failures, missing itemsets, and artificial itemsets are used to evaluate the performance of data sanitization. for data distortion [28, 31, 32] of sensitive itemsets in PPDM. The relationships between the side effects and mined itemsets of the original database and sanitized one are shown in Figure 1.

In Figure 1, *F* represents the frequent itemsets mined from the original database, *F*′represents the frequent itemsets mined from the sanitized database, and *S* represents the sensitive itemsets that should be hidden. The *α* part is concerned as hiding failures that fail to hide the sensitive itemsets. Thus, *α* is the intersection of *S* and *F*′ ( = *S*∩*F*′). *β* part is concerned as missing itemsets that mistakenly to delete the nonsensitive frequent rules. Thus, *β* is the difference between *F*, *S*, and *F*′ ( = *F* − *S* − *F*′). *γ* part is concerned as artificial itemsets which is unexpectedly generated. Thus, *γ* is the difference between *F*′ and *F* ( = *F*′ − *F*). In PPDM, it is intuitive to delete transactions with sensitive itemsets in the sanitization process. In this paper, *α*, *β*, and *γ* with adjustable weights are considered to evaluate whether the processed transactions are required to be deleted. Besides the above side effects, the number of deleted transactions or items is also a criterion to evaluate the data distortion [32, 33].

## 3. Proposed Hiding-Missing-Artificial Utility Algorithm

### 3.1. Definition of Formulas

Data sanitization is the most common way to protect sensitive knowledge from disclosure in PPDM. To avoid the side effects of hiding failures, missing itemsets, and artificial itemsets, minimal distortion of the databases is thus necessary. In this paper, a hiding-missing-artificial utility (HMAU) algorithm is proposed to hide sensitive itemsets through transaction deletion. Three dimensions of hiding failure dimension (HFD), missing itemset dimension (MID), and artificial itemset dimension (AID) are thus concerned to evaluate whether the transactions are required to be deleted for hiding the sensitive itemsets. The transactions with any of the sensitive itemset are first evaluated by the designed algorithm to find the minimal HMAU values among transactions, The transaction with minimal HMAU value will be directly removed from the database. The procedure is thus repeated until all sensitive itemsets are hidden. In order to avoid exposing the already hidden sensitive itemsets again, the minimum count is dynamically updated during the deletion procedure.

The value of each dimension is set from 0 to 1 (0 < value ≤ 1). In the proposed formulas, the differences between minimum support threshold and the frequencies of the sensitive itemsets are thus considered to evaluate whether the transactions are required to be deleted instead of only the presence of the itemsets in the transactions.

First, the HFD is used to evaluate the hiding failures of each processed transaction in the sanitization process. When a processed transaction *T*_*k*_ contains a sensitive itemset *hs*_*x*_, the HFD value of the processed transaction is calculated as

(1) $$\begin{array}{c} {\text{HFD}}^{k}\left({\text{hs}}_{x}\right)=\frac{{\text{MAX}}_{\text{HS}}-\text{freq}\left({\text{hs}}_{x}\right)+1}{{\text{MAX}}_{\text{HS}}-\left\lceil \left|D\right|\times \lambda \right\rceil +1}, \end{array}$$

where *λ* is defined as the percentage of the minimum support threshold, sensitive itemset hs_*x*_ is from the set of sensitive itemsets HS, MAX_HS_ is the maximal count of the sensitive itemsets in the set of sensitive itemsets HS, |*D*| is the number of transactions in the original database *D*, and freq(hs_*x*_) is the occurrence frequency of the sensitive itemset hs_*x*_.

Second, the MID is used to evaluate the itemsets of each processed transaction in the sanitization process. When a processed transaction *T*_*k*_ contains a frequent itemset fi_*x*_, the MID value of the processed transaction is calculated as

(2) $$\begin{array}{c} {\text{MID}}^{k}\left({\text{fi}}_{x}\right)=\frac{{\text{MAX}}_{\text{FI}}-\text{freq}\left({\text{fi}}_{x}\right)+1}{{\text{MAX}}_{\text{FI}}-\left\lceil \left|D\right|\times \lambda \right\rceil +1}, \end{array}$$

where an itemset fi_*x*_ is a frequent itemset from the set of large (frequent) itemsets FI, MAX_FI_ is the maximal count of the large itemsets in the set of FI, and freq(fi_*x*_) is the occurrence frequency of the large itemset fi_*x*_.

Third, the AID is used to evaluate the artificial itemsets of each processed transaction in the sanitization process. In AID, only the small 1-itemsets are considered in the sanitization process since it is a nontrivial task to keep all infrequent itemsets. When a processed transaction *T*_*k*_ contains a small 1-itemset si_*x*_, the AID value of the processed transaction is calculated as

(3) $$\begin{array}{c} {\text{AID}}^{k}\left({\text{si}}_{x}\right)=\frac{\text{freq}\left({\text{si}}_{x}\right)-{\text{MIN}}_{{\text{SI}}^{1}}+1}{\left\lceil \left|D\right|\times \lambda \right\rceil -{\text{MIN}}_{{\text{SI}}^{1}}}, \end{array}$$

where a small 1-itemset si_*x*_ is from the set of small 1-itemsets SI^1^, MIN_SI^1^_ is the minimal count of the small 1-itemsets in the set of SI^1^, and freq(si_*x*_) is the occurrence frequency of the small 1-itemset si_*x*_.

In this paper, a risky bound is designed to speed up the execution time of the proposed HMAU algorithm by avoiding the evaluation of all large itemsets and small 1-itemsets by considering MID and AID. A parameter *μ* is set as the percentage used to find the upper and lower boundaries of the minimum support threshold. Only the large itemsets and infrequent 1-itemsets within the boundaries are used to determine whether the processed transactions are required to be deleted. For the large itemsets, the minimum support threshold is set as the lower boundary, and the upper boundary is set as

(4) $$\begin{array}{c} \text{freq}\left({\text{fi}}_{j}\right)\leq \left\lceil \left\lceil \left|D\right|\times \lambda \right\rceil \times \left(1+\mu \right)\right\rceil , \end{array}$$

where |*D*| is the number of transactions in the original database *D*, *λ* is the minimum support threshold, *μ* is the risky bound, and freq(fi_*j*_) is the occurrence frequency of the large itemset fi_*j*_.

For small 1-itemsets, the minimum support threshold is set as the upper boundary, and the lower boundary is set as

(5) $$\begin{array}{c} \text{freq}\left({\text{si}}_{a}\right)\geq \left\lfloor \left\lceil \left|D\right|\times \lambda \right\rceil \times \left(1-\mu \right)\right\rfloor , \end{array}$$

where freq(si_*a*_) is the occurrence frequency of the small 1-itemset si_*a*_.

**Figure 2:**
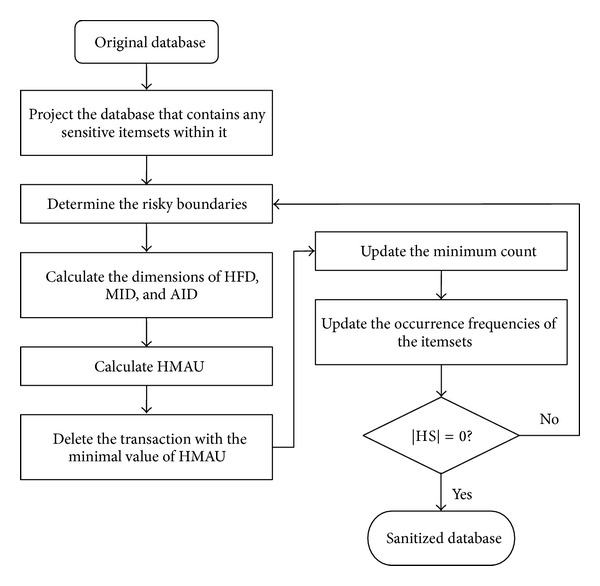
Flowchart of the proposed HMAU algorithm.

The flowchart of the proposed HMAU algorithm is depicted in Figure 2.

### 3.2. Notation

See Table 1.

Details of the proposed HMAU algorithm are illustrated as follows.

*Proposed HMAU Algorithm*. 

*Input.* This includes an original database *D*, a minimum support threshold ratio *λ*, a risky bound *μ*, a set of large (frequent) itemsets FI = {fi_1_, fi_2_, …, fi_*p*_}, a set of small (nonfrequent) 1-itemsets SI^1^ = {si_1_, si_2_, …, si_*q*_}, and a set of sensitive itemsets to be hidden HS = {hs_1_, hs_2_, …, hs_*r*_}.

*Output.* This includes a sanitized database D* with no sensitive information. 

*Step  1*. Select the transactions to form a projected database *D*′, where each transaction *T*_*k*_ in *D*′ consists of sensitive itemsets hs_*i*_ within it, where 1 ≤ *i* ≤ *r*. 

*Step  2*. Process each frequent itemset fi_*j*_ in the set of FI to determine whether its frequency satisfies the condition freq(fi_*j*_) ≤ ⌈⌈|*D* | ×*λ*⌉ × (1 + *μ*)⌉, where |*D*| is the number of transactions in the original database *D* and freq(fi_*j*_) is the occurrence frequency of the large itemset fi_*j*_. Put the fi_*j*_ that do not satisfy the condition into the set of FI_tmp_. 

*Step  3*. Process each small 1-itemset si_*a*_ in the set of SI^1^ to determine whether its frequency satisfies the condition freq(si_*a*_) ≥ ⌊⌈|*D* | ×*λ*⌉ × (1 − *μ*)⌋, where freq(si_*a*_) is the occurrence frequency of the small 1-itemset si_*a*_. Put the si_*a*_ that do not satisfy the condition into the set of SI_tmp_^1^. 

*Step  4*. Calculate the maximal count (MAX_HS_) of the sensitive itemsets hs_*i*_ in the set of HS as

(6) $$\begin{array}{c} \text{MA}{\text{X}}_{\text{HS}}=\max\left\{\text{freq}\left({\text{hs}}_{i}\right),\;\;\forall {\text{hs}}_{i},1\leq i\leq r\right\}, \end{array}$$

where freq(hs_*i*_) is the occurrence frequency of the sensitive itemset hs_*i*_ in the set of HS.

*Step  5*. Calculate the HFD of each transaction *T*_*k*_. Do the following substeps. 

*Substep  5.1*. Calculate the HFD of each sensitive itemset hs_*i*_ within *T*_*k*_ as

(7) $$\begin{array}{c} {\text{HFD}}^{k}\left({\text{hs}}_{i}\right)=\frac{{\text{MAX}}_{\text{HS}}-\text{freq}\left({\text{hs}}_{i}\right)+1}{{\text{MAX}}_{\text{HS}}-\left\lceil \left|D\right|\times \lambda \right\rceil +1}. \end{array}$$

*Substep  5.2*. Sum the HFDs of sensitive itemsets hs_*i*_ within *T*_*k*_ as

(8) $$\begin{array}{c} {\text{HFD}}^{k}=\frac{1}{\sum_{i=1}^{r}{\text{HFD}}^{k}\left({\text{hs}}_{i}\right)+1}. \end{array}$$

*Substep  5.3*. Normalize the HFD^*k*^ for all transactions *T*_*k*_ in *D*′.

*Step  6*. Calculate the maximal count (MAX_FI_) of the large itemsets fi_*j*_ in the set of FI as

(9) $$\begin{array}{c} {\text{MAX}}_{\text{FI}}=\max\left\{\text{freq}\left({\text{fi}}_{j}\right),\;\;\forall {\text{fi}}_{j},1\leq j\leq p\right\}. \end{array}$$

*Step  7*. Calculate the MID of each transaction *T*_*k*_. Do the following substeps. 

*Substep  7.1*. Calculate the MID of each large itemset within *T*_*k*_ as

(10) $$\begin{array}{c} {\text{MID}}^{k}\left({\text{fi}}_{j}\right)=\frac{{\text{MAX}}_{\text{FI}}-\text{freq}\left({\text{fi}}_{j}\right)+1}{{\text{MAX}}_{\text{FI}}-\left\lceil \left|D\right|\times \lambda \right\rceil +1}. \end{array}$$

*Substep  7.2*. Sum the MIDs of large itemsets fi_*j*_ within *T*_*k*_ as

(11) $$\begin{array}{c} {\text{MID}}^{k}=\overset{p}{\underset{j=1}{\sum }}{\text{MID}}^{k}\left({\text{fi}}_{j}\right). \end{array}$$

*Substep  7.3*. Normalize the MID^*k*^ for all transactions *T*_*k*_ in *D*′. 

*Step  8*. Calculate the minimal count (MIN_SI^1^_) of the small 1-itemsets si_*a*_ in the set of SI^1^ as

(12) $$\begin{array}{c} {\text{MIN}}_{\text{S}{\text{I}}^{1}}=\min\left\{\text{freq}\left({\text{si}}_{a}\right),\;\;\forall {\text{si}}_{a},1\leq a\leq q\right\}. \end{array}$$

*Step  9*. Calculate the AID of each transaction *T*_*k*_. Do the following substeps. 

*Substep  9.1*. Calculate the AID of each small 1-itemset within *T*_*k*_ as

(13) $$\begin{array}{c} {\text{AID}}^{k}\left({\text{si}}_{a}\right)=\frac{\text{freq}\left({\text{si}}_{a}\right)-{\text{MIN}}_{\text{S}{\text{I}}^{1}}+1}{\left\lceil \left|D\right|\times \lambda \right\rceil -{\text{MIN}}_{\text{S}{\text{I}}^{1}}}. \end{array}$$

*Substep  9.2*. Sum the AIDs of small 1-itemsets si_*a*_ within *T*_*k*_ as

(14) $$\begin{array}{c} {\text{AID}}^{k}=\frac{1}{\sum_{a=1}^{q}{\text{AID}}^{k}\left({\text{si}}_{a}\right)+1}. \end{array}$$

*Substep  9.3*. Normalize the AID^*k*^ for all transactions *T*_*k*_ in *D*′. 

*Step  10*. Calculate the HMAU for HFD, MID, and AID of each transaction *T*_*k*_ as

(15) $$\begin{array}{c} {\text{HMAU}}^{k}=w_{1}\times {\text{HFD}}^{k}+w_{2}\times {\text{MID}}^{k}+w_{3}\times {\text{AID}}^{k}, \end{array}$$

where *w*_1_, *w*_2_, and *w*_3_ are the predefined weights by users. 

*Step  11*. Remove transaction *T*_*k*_ with min⁡{HMAU^*k*^, ∀ *T*_*k*_, 1 ≤ *k* ≤ | *D*′|} value. 

*Step  12*. Update the minimum count ( = ⌈|*D* | ×*λ*⌉**) **of sanitized database.

*Step  13*. Update the occurrence frequencies of all sensitive itemsets in the sets of HS and HS_tmp_. Put hs_*i*_ into the set of HS_tmp_ if freq(hs_*i*_) < minimum count ( = ⌈|*D* | ×*λ*⌉), and put hs_*i*_ into the set of HS otherwise. 

*Step  14*. Update the occurrence frequencies of all large itemsets in the sets of FI and FI_tmp_. Put fi_*j*_ into the set of FI_tmp_ if freq(fi_*j*_) < minimum count ( = ⌈|*D* | ×*λ*⌉), and put fi_*j*_ into the set of FI otherwise. 

*Step  15*. Update the occurrence frequencies of all small 1-itemsets in the sets of SI^1^ and SI_tmp_^1^. Put si_*a*_ into the set of SI_tmp_^1^ if freq(si_*a*_) ≥ minimum count ( = ⌈|*D* | ×*λ*⌉), and put si_*a*_into the set of SI^1^ otherwise. 

*Step  16*. Repeat Step  2 to Step  15 until the set of HS is empty (|HS | = 0). 

## 4. An Illustrated Example

In this section, an example is used to illustrate the proposed algorithm step by step. Consider a database with 10 transactions (tuples) and 6 items (denoted as *a* to *f*) shown in Table 2. Each transaction can be considered a set of purchased items in a trade. The minimum support threshold is initially set at 40%, and the risky bound is set at 10%. A set of sensitive itemsets, HS = {*be* : 6, *a* *be* : 4}, is considered to be hidden by the sanitization process.

Based on an Apriori-like approach [3], the large (frequent) itemsets and small 1-itemsets are mined. The results are, respectively, shown in Tables 3 and 4.

The proposed algorithm then proceeds as follows to sanitize the database for hiding all sensitive itemsets in HS.

**Step 1**

The transactions in *D* are selected with any of the sensitive itemsets in HS. In this example, the transactions 1, 3, 6, 7, 8, and 10 are selected to form the database shown in Table 5.

**Step 2**

The frequent itemsets in FI are processed to check whether the condition is satisfied, which is calculated as freq(fi_*j*_) ≤ ⌈⌈10 × 0.4⌉ × (1 + 0.1)⌉ (= freq(fi_*j*_) ≤ 5). The itemsets {*a*, *ab*, *ae*, *bc*, *bc* *e*} satisfy the condition and are kept in FI; the remaining itemsets, {*b*, *c*, *e*, *ce*}, are put into the set of FI_tmp_.

**Step 3**

The infrequent 1-itemsets in SI^1^ are then processed to check whether the condition is satisfied, which is calculated as freq(si_*a*_) ≥ ⌊⌈10 × 0.4⌉ × (1 − 0.1)⌋ (= freq(si_*a*_) ≥ 3). The itemset {*d*} satisfies the condition and is kept as SI^1^; the other itemset, {*f*}, is put into the set of SI_tmp_^1^.

**Step 4**

The maximal count (MAX_HS_) among the sensitive itemsets in the set of HS is then calculated. In this example, the maximal count of the sensitive itemsets {*be*} and {*a* *be*} is calculated as MAX_HS_ = max⁡{6, 4} = 6.

**Step 5**

The HFD of each transaction is calculated to evaluate the side effects of hiding failures of the processed transaction. In this example, transaction 7 is used to illustrate the following steps. According to formula (1), the HFD is calculated as HFD^7^(*be*) = (6 − 6 + 1)/(6 − 4 + 1) = 0.33 and HFD^7^(*a* *be*) = (6 − 4 + 1)/(6 − 4 + 1) = 1. The HFD of transaction 7 is calculated as HFD^7^ = 1/(0.33 + 1 + 1) = 0.43. The other transactions are processed in the same way. The results are shown in Table 6.

The HFDs for all transactions are then normalized as shown in Table 7.

**Step 6**

The maximal count (MAX_FI_) among the large itemsets in the set of FI is then calculated. In this example, the large itemsets are {*a*, *ab*, *ae*, *bc*, *bc* *e*}, and the MAX_FI_ is calculated as MAX_FI_ = max⁡{5, 5, 4, 5, 5} (=5).

**Step 7**

The MID of each transaction is calculated to evaluate the side effects of missing itemsets of the processed transaction. The frequent item {*a*} in transaction 7 is used as an example to illustrate the steps. According to formula (2), the MID of the item {*a*} is calculated as MID^7^(*a*) = (5 − 5 + 1)/(5 − 4 + 1) = 0.5. The other frequent itemsets *ab*, *ae*, *bc*, and *bc* *e* in transaction 7 are calculated in the same way, with MID^7^(*ab*) = 0.5, MID^7^(*ae*) = 1, MID^7^(*bc*) = 0.5, and MID^7^(*bc* *e*) = 0.5. The MID of transaction 7 is then calculated as MID^7^ = 0.5 + 0.5 + 1 + 0.5 + 0.5  ( = 3). The other transactions are processed in the same way. The results are shown in Table 8.

The MIDs for all transactions are then normalized as shown in Table 9.

**Step 8**

The minimal count (MIN_SI^1^_) among the small 1-itemsets in the set of SI^1^ is then calculated. In this example, the small 1-itemset has only {*d*}, and the minimal count of the small 1-itemset is calculated as MIN_SI^1^_ = min{3} = 3.

**Step 9**

The AID of each transaction is calculated to evaluate the side effects of artificial itemsets of the processed transaction. Small 1-itemset {*d*} in transaction 7 is used as an example to illustrate the steps. According to formula (3), the AID of the small 1-itemset {*d*} is calculated as AID^7^(*d*) = (3 − 3 + 1)/(4 − 3) = 1; since there is only one itemset in the set of SI^1^, no other calculations are necessary. The AID of transaction 7 is calculated as AID^7^ = 1/(1 + 1) = 0.5. The other transactions are processed in the same way. The results are shown in Table 10.

The AIDs for all transactions are then normalized as shown in Table 11.

**Step 10**

The three dimensions for evaluating the selected transactions are then organized as in Table 12. The weights of hiding failures, missing itemsets, and artificial itemsets are, respectively, set to 0.5, 0.4, and 0.1. Note that these values can be defined by users to decide the importance among the dimensions. In this example, the HMAU of transaction 7 is calculated as

(16) $$\begin{array}{c} {\text{HMAU}}^{7}=0.5\times 0.57+0.4\times 1+0.1\times 0.5\;\;\left(=0.735\right). \end{array}$$

The other transactions are processed in the same way. The results are shown in the last column of Table 12.

**Step 11**

The selected transactions in Table 12 are then evaluated to find a transaction with the minimal HMAU value. In this example, transaction 8 has the minimal value and is directly removed from Table 12.

**Step 12**

Transaction 8 is deleted in the dataset in this example. The minimum count is updated as ⌈|10 − 1 | ×0.4⌉ (= 4).

**Step 13**

The occurrence frequencies of all sensitive itemsets in the sets of HS and HS_tmp_ are, respectively, updated. Since the original database with transaction 8 consisted of the sensitive itemsets {*be*, *a* *be*}, which was deleted in Step 11, the counts of {*be*, *a* *be*} in the set of HS are, respectively, updated as {*be*} (= 6 − 1) (= 5) and {*a* *be*} (= 4 − 1) (= 3). In this example, the set of HS_tmp_ is empty, so there is nothing to be done in this step. After the updating process, the itemset {*a* *be*} is put into the set of HS_tmp_ since its count is below the minimum count (3 < 4).

**Step 14**

The occurrence frequencies of all large itemsets in the sets of FI and FI_tmp_ are, respectively, updated. Since the original database with transaction 8 consisted of the large itemsets {*a*, *b*, *e*, *ab*, *ae*}, which was deleted in Step 11, the counts of {*a*, *b*, *e*, *ab*, *ae*} in the set of FI and FI_tmp_ are, respectively, updated as {*a*} (= 5 − 1) (= 4), {*b*} (= 7 − 1) (= 6), {*e*} (= 8 − 1) (= 7), {*ab*} (= 5 − 1) (= 4), and {*ae*} (= 4 − 1) (= 3). After the updating process, the itemset {*ae*} is put into the set of FI_tmp_ since its count is below the minimum count (3 < 4).

**Step 15**

The occurrence frequencies of all small 1-itemsets in the sets of SI^1^ and SI_tmp_^1^ are, respectively, updated. Since the original database with transaction 8 did not consist of any of the small 1-itemsets in SI^1^ and SI_tmp_^1^, nothing is done in this step.

**Step 16**

In this example, the sensitive itemset {*a* *be*} is already hidden, but the occurrence frequency of sensitive itemset {*be*} is larger than the minimum count. Steps 2 to 15 are repeated until the set of sensitive itemsets HS is empty (|HS | = 0). After all Steps are processed, the sanitized database is obtained as shown in Table 13.

Comparing the original database and the sanitized one, transactions 1, 3, 6, and 8 are removed from the original database, and the minimum count is updated as 3. The updated frequent itemsets of the sanitized database are shown in Table 14.

Comparing the large itemsets in Table 3, the sensitive itemsets {*be*} and {*a* *be*} are hidden and no artificial itemset is generated. Three itemsets, {*ae*, *bc*, *bc* *e*}, are, however, missing itemsets of the sanitized database. In this example, the side effects of hiding failures, missing itemsets, and artificial itemsets are 0, 3, and 0, respectively.

## 5. Experimental Results

Experiments are conducted to show the performance of the proposed HMAU algorithm compared to that of the aggregate algorithm [23] for hiding sensitive itemsets through transaction deletion. The experiments were coded in C++ and performed on a personal computer with an Intel Core i7-2600 processor at 3.40 GHz and 4 GB of RAM running 64-bit Microsoft Windows 7. The real database BMS-WebView-1 [34] and a synthetic database (T7I7N200D20K) [35] from IBM data generator in which *T* symbolizes the average length of the transactions, *I* symbolizes the average maximum size of frequent itemsets, *N* symbolizes the number of differential items, and *D* symbolizes the size of database were used in the experiments. The details of the two databases are shown in Table 15.

For the BMS-WebView-1 database, the minimum support thresholds were, respectively, set at 1% and 2% to evaluate the performance of the proposed approach, and the percentages of sensitive itemsets were sequentially set from 5% to 25% of the number of frequent itemsets in 5% increments. In the experiments, the weights of HFD, MID, and AID in the proposed algorithm were, respectively, set at 0.5, 0.4, and 0.1.

For the T7I7N200D20K database, the minimum support thresholds were, respectively, set at 1.5% and 3%, and the percentages of sensitive itemsets were sequentially set at 2.5% to 12.5% of the number of frequent itemsets in 2.5% increments. In the experiments, the weights of HFD, MID, and AID in the proposed algorithm were, respectively, set at 0.5, 0.4, and 0.1.

### 5.1. Comparisons of Execution Time

**Figure 3:**
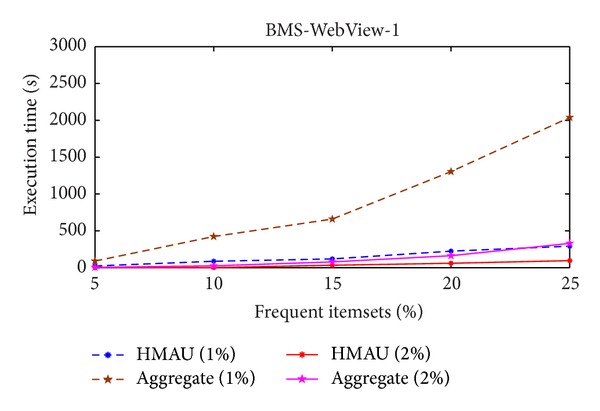
Comparison of execution time in BMS-WebView-1 database.

Figure 3 shows the execution time of two algorithms in BMS-Web-View-1 database. Different minimum support thresholds of two algorithms are then compared in various sensitivity percentages of the frequent itemsets.

**Figure 4:**
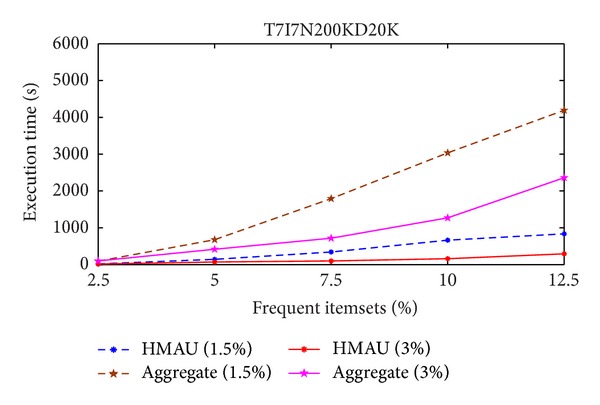
Comparison of execution time in T7I7N200D20K database.

The execution time of the proposed HMAU algorithm is faster than those of the aggregate algorithm whether the minimum support threshold is set at 1% or 2%. Experiment is then conducted in T7I7N200D20K database and the results are shown in Figure 4.

From Figures 3 and 4, it is obvious to see that the proposed HMAU algorithm is faster than those of the aggregate method in two different databases.

### 5.2. Comparisons of Number of Deleted Transactions

**Figure 5:**
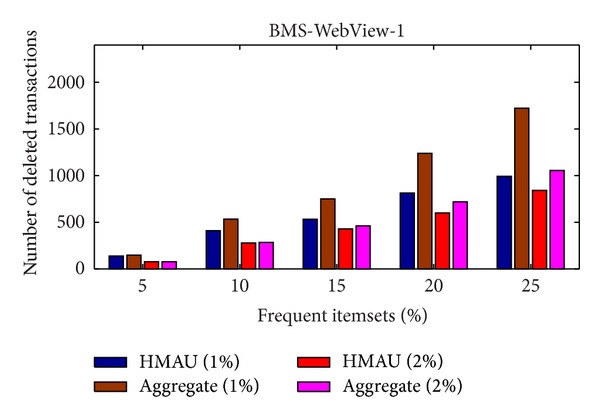
Comparison of number of deleted transactions in BMS-WebView-1 database.

Experiments were also conducted to evaluate the number of deleted transactions of the proposed algorithm in two different databases. For the BMS-WebView-1 database, the results are shown in Figure 5.

**Figure 6:**
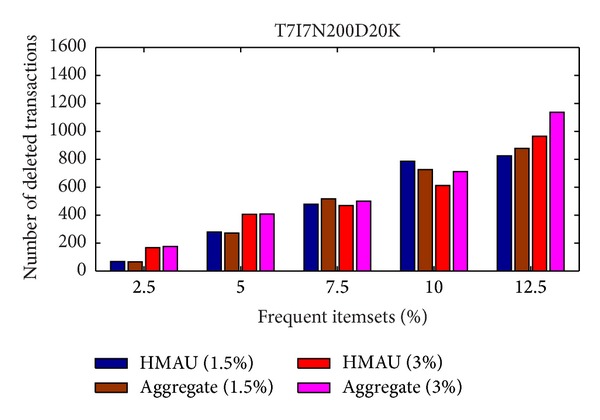
Comparison of number of deleted transactions in T7I7N200D20K database.

From Figure 5, it is obvious to see that the proposed HMAU algorithm deletes fewer transactions than the aggregate algorithm whether the minimum support threshold is set at 1% or 2% in BMS-WebView-1 database, thus achieving lower data distortion. For the T7I7N200D20K database, the results are shown in Figure 6.

From Figure 6, it is obvious to see that when the sensitive itemsets were set at 10% of the frequent itemsets with 1.5% minimum support threshold in T7I7N200D20K database, the proposed HMAU algorithm produced more transactions to be deleted for hiding sensitive itemsets. Since the proposed HMAU algorithm considers the three dimensions together, the selected transactions for deletion may consist of fewer large transactions rather than many sensitive itemsets.

### 5.3. Comparisons of Side Effects

Three side effects are then compared to show the performance of the proposed algorithm in two different databases.

The side effects of hiding failures, missing itemsets, and artificial itemsets are, respectively, symbolized as *α*, *β*, and *γ*. In Table 16, it can be seen that when the minimum support threshold was set at 1%, the proposed HMAU algorithm produces no side effects whereas the aggregate algorithm produces some artificial itemsets since the criteria of artificial itemsets are not considered in aggregate algorithm. Both the two algorithms produce no side effects when the minimum support threshold was set at 2%. The results to evaluate the side effects of the proposed HMAU algorithm in T7I7N200D20K database are shown in Table 17.

From Table 17, it is obvious to see that when the minimum support threshold was set at 1.5%, the proposed HMAU algorithm produces fewer artificial itemsets and missing itemsets than the aggregate algorithm for various sensitivity percentages of the frequent itemsets. The proposed HMAU algorithm produces no side effects at 3% minimum support threshold whereas the aggregate algorithm produces some artificial itemsets.

To summarize the above results for BMS-WebView-1 and T7I7N200D20K databases, the proposed HMAU algorithm outperforms the aggregate algorithm in terms of the execution time, the number of deleted transactions, and the number of side effects.

## 6. Conclusion and Future Works

In this paper, the HMAU algorithm is proposed for hiding sensitive itemsets in data sanitization process by reducing the side effects through transaction deletion. The formulas of three dimensions as HFD, MID, and AID are defined to evaluate the correlation between the processed transactions and side effects. The weights of three evaluation dimensions of HFD, MID, and AID can be set by users' interests. In the experiments, both the real dataset and synthetic dataset are used to, respectively, evaluate the performances of the two proposed algorithms. Experimental results showed that the proposed HMAU algorithm outperforms the aggregate algorithm in terms of execution time, number of deleted transactions, and number of side effects.

In the future, the sensitive itemsets to be hidden can be extended to the sensitive association rules to be hidden. More considerations are necessary to be concerned to decrease not only the supports of sensitive itemsets but also the confidence of sensitive association rules. Other distortion approaches such as the noise addition and data modification are also the important issues to hide the sensitive information in PPDM.

## Figures and Tables

**Table 1:** The notations used in the proposed HMAU algorithm are described below.

*D*	An original database, *D* = {*T*_1_, *T*_2_,…, *T*_*x*_,…, *T*_*n*_}, in which each *T*_*x*_ represents a transaction
*D*′	A projected database, *D*′ = {*T*_1_, *T*_2_,…, *T*_*y*_,…, *T*_*k*_}, in which each *T*_*y*_ contains sensitive itemsets
D*	A sanitized database, from which no sensitive information can be mined
*λ*	The minimum support threshold ratio
*μ*	The risky bound parameter, using which the itemsets within the boundary are used to evaluate the processed transactions
FI	The set of frequent itemsets FI = {fi_1_, fi_2_,…, fi_*j*_,…, fi_*p*_} in *D*, in which each itemset fi_*j*_ is larger than or equal to the minimum support threshold
fi_*j*_	A frequent itemset
SI^1^	The set of infrequent 1-itemsets SI^1^ = {si_1_, si_2_,…, si_*a*_,…, si_*q*_} in *D*, in which each itemset *si*_*a*_ is below the minimum support threshold
si_*a*_	A small (infrequent) 1-itemset
HS	A set of sensitive itemsets HS = {hs_1_, hs_2_,…, hs_*i*_,…, hs_*r*_}, in which each element represents an itemset that should be hidden in the original database
hs_*i*_	A sensitive itemset
HS_tmp_	The temporary set of sensitive itemsets outside the boundary
FI_tmp_	The temporary set of large itemsets outside the boundary
SI_tmp_^1^	The temporary set of small 1-itemsets outside the boundary
HFD	The hiding failure dimension used to consider the side effects of hiding failures
MID	The missing itemset dimension used to consider the side effects of missing itemsets
AID	The artificial itemset dimension used to consider the side effects of artificial itemsets
HFD^*k*^(hs_*i*_)	The value of the sensitive itemset hs_*i*_ in transaction *T*_*k*_
MID^*k*^(fi_*j*_)	The value of the large itemset fi_*j*_ in transaction *T*_*k*_
AID^*k*^(si_*a*_)	The value of the small 1-itemset si_*a*_ in transaction *T*_*k*_
MAX_HS_	The maximal count of the sensitive itemsets in the set of *HS*
freq(hs_*i*_)	The occurrence frequency of the sensitive itemset hs_*i*_ in the set of HS
MAX_FI_	The maximal count of the large itemsets in the set of FI
freq(*fi*_*j*_)	The occurrence frequency of the large itemset fi_*j*_
MIN_SI^1^_	The minimal count of the small 1-itemsets in the set of SI^1^
freq(si_*a*_)	The occurrence frequency of the small 1-itemset si_*a*_
*w*_*b*_	The weights for HFD, MID, and AID, in which 0 < *w*_*b*_ ≤ 1
HMAU	The utility value used to determine whether the processed transactions should be deleted

**Table 2:** Original database.

TID	Item
*T*_1_	*a, b, c, e *
*T*_2_	*e *
*T*_3_	*b, c, e, f *
*T*_4_	*d, f *
*T*_5_	*a, b, d *
*T*_6_	*b, c, e *
*T*_7_	*a, b, c, d, e *
*T*_8_	*a, b, e *
*T*_9_	*c, e *
*T*_10_	*a, b, c, e *

**Table 3:** Large itemsets.

Large 1-itemset	Count	Large 2-itemset	Count	Large 3-itemset	Count
*a *	5	*ab *	5	*abe *	4
*b *	7	*ae *	4	*bce *	5
*c *	6	*bc *	5		
*e *	8	*be *	6		
		*ce *	6		

**Table 4:** Small 1-itemsets.

Small 1-itemset	Count
*d *	3
*f *	2

**Table 5:** Projected database *D*′.

TID	Item
*T*_1_	*a, b, c, e *
*T*_3_	*b, c, e, f *
*T*_6_	*b, c, e *
*T*_7_	*a, b, c, d, e *
*T*_8_	*a, b, e *
*T*_10_	*a, b, c, e *

**Table 6:** Hiding failure dimension for all transactions.

TID	HFD
*T*_1_	0.43
*T*_3_	0.75
*T*_6_	0.75
*T*_7_	0.43
*T*_8_	0.43
*T*_10_	0.43

**Table 7:** Normalization of HFDs for all transactions.

TID	HFD
*T*_1_	0.57
*T*_3_	1
*T*_6_	1
*T*_7_	0.57
*T*_8_	0.57
*T*_10_	0.57

**Table 8:** Missing itemset dimension for all transactions.

TID	MID
*T*_1_	3
*T*_3_	1
*T*_6_	1
*T*_7_	3
*T*_8_	2
*T*_10_	3

**Table 9:** Normalization of MIDs for all transactions.

TID	MID
*T*_1_	1
*T*_3_	0.33
*T*_6_	0.33
*T*_7_	1
*T*_8_	0.67
*T*_10_	1

**Table 10:** Artificial itemset dimension for all transactions.

TID	AID
*T*_1_	1
*T*_3_	1
*T*_6_	1
*T*_7_	0.5
*T*_8_	1
*T*_10_	1

**Table 11:** Normalization of AIDs for all transactions.

TID	AID
*T*_1_	1
*T*_3_	1
*T*_6_	1
*T*_7_	0.5
*T*_8_	1
*T*_10_	1

**Table 12:** Three dimensions of each transaction in projected database.

TID	HFD	MID	AID	HMAU
*T*_1_	0.57	1	1	0.785
*T*_3_	1	0.33	1	0.733
*T*_6_	1	0.33	1	0.733
*T*_7_	0.57	1	0.5	0.735
*T*_8_	0.57	0.67	1	0.652
*T*_10_	0.57	1	1	0.785

**Table 13:** Sanitized database.

TID	Item
*T*_2_	*e*
*T*_4_	*d*, *f*
*T*_5_	*a*, *b*, *d*
*T*_7_	*a*, *b*, *c*, *d*, *e*
*T*_9_	*c*, *e*
*T*_10_	*a*, *b*, *c*, *e*

**Table 14:** Large itemsets of the sanitized database.

Large 1-itemset	Count	Large 2-itemset	Count
*a*	3	*ab*	3
*b*	3	*ce*	3
*c*	3		
*e*	4		

**Table 15:** Details of real and synthetic databases.

Dataset	Number of transactions	Number of items	Maximum transaction size	Average transaction size
BMS-WebView-1	59,602	497	267	2.5
T7I7N200D20K	15,351	200	26	8.7

**Table 16:** Comparison of side effects in BMS-WebView-1 database.

Sensitive percentage of FIs (minimum support threshold)	HMAU			Aggregate		
	*α*	*β*	*γ*	*α*	*β*	*γ*
5% (1%)	0	0	0	0	0	0
10% (1%)	0	0	0	0	0	1
15% (1%)	0	0	0	0	0	1
20% (1%)	0	0	0	0	0	3
25% (1%)	0	0	0	0	0	2

5% (2%)	0	0	0	0	0	0
10% (2%)	0	0	0	0	0	0
15% (2%)	0	0	0	0	0	0
20% (2%)	0	0	0	0	0	0
25% (2%)	0	0	0	0	0	0

**Table 17:** Comparison of side effects in T7I7N200D20K database.

Sensitive percentage of FIs (minimum support threshold)	HMAU			Aggregate		
	*α*	*β*	*γ*	*α*	*β*	*γ*
2.5% (1.5%)	0	0	1	0	1	2
5% (1.5%)	0	0	0	0	3	4
7.5% (1.5%)	0	0	3	0	3	7
10% (1.5%)	0	0	0	0	2	6
12.5% (1.5%)	0	0	1	0	3	6

2.5% (3%)	0	0	0	0	0	0
5% (3%)	0	0	0	0	0	2
7.5% (3%)	0	0	0	0	0	1
10% (3%)	0	0	0	0	0	1
12.5% (3%)	0	0	0	0	0	2
